# High-resolution manometry in patients with and without globus pharyngeus and/or symptoms of laryngopharyngeal reflux

**DOI:** 10.1186/s12876-017-0666-x

**Published:** 2017-10-23

**Authors:** Heyan Ding, Zhijun Duan, Dong Yang, Zhifeng Zhang, Lixia Wang, Xiaoyu Sun, Yiwen Yao, Xue Lin, Hang Yang, Shan Wang, Jiande D. Z. Chen

**Affiliations:** 1 Neurogastroenterology and Motility Center of China-US Cooperation, Second Gastroenterology Department, First Affiliated Hospital of Dalian Medical University, Dalian, 116011 China; 2 Otolaryngology Department, First Affiliated Hospital of Dalian Medical University, Dalian, 116011 China; 3 Division of Gastroenterology and Hepatology, Johns Hopkins Medicine, Baltimore, MD 21224 USA

**Keywords:** Globus pharyngeus, Upper esophageal sphincter (UES), Lower esophageal sphincter (LES), Distal esophageal contraction integral (DCI)

## Abstract

**Background:**

Globus pharyngeus is common and has a low cure rate. Its etiology is complex and reported to be associated with laryngopharyngeal reflux (LPR). However, some patients with globus do not exhibit any reflux symptoms or respond to proton pump inhibitors (PPIs) treatments. The purpose of this study was to clarify the related risk factors of these patients with a final objective of improving the curative effect.

**Methods:**

Forty two patients afflicted with globus pharyngeus (G group) and 38 patients without globus pharyngeus (NG group) were included in this study. According to the laryngopharyngeal Reflux Symptom Index and the response to PPIs treatments, the patients were further divided into reflux groups (G-R, NG-R) and non-reflux groups (G-NR, NG-NR). High Resolution Manometry (HRM) was performed to assess esophageal motility. Questionnaires, including categories such as life exposure factors, were conducted.

**Results:**

*a)* The average resting and residual pressures of the upper esophageal sphincter (UES) in the G-NR group was higher than in the NG-NR and NG-R groups (*P* < 0.05). *b)* The average resting and residual pressures of the lower esophageal sphincter showed no differences between the G-NR group and the NG-NR group (*P* > 0.05). *c)* The esophageal distal contractile integral score of the G-NR group was not different from the NG-NR group (*P* > 0.05). *d)* Compared to the NG-NR group, the G-NR group showed higher incidence of stress, smoking, drinking, high salt and anxiety (*P* < 0.05).

**Conclusions:**

Globus pharyngeus without LPR may occur due to high UES pressure. Stress, smoking, alcoholic drinking, high salt and anxiety may be its risk factors.

**Electronic supplementary material:**

The online version of this article (10.1186/s12876-017-0666-x) contains supplementary material, which is available to authorized users.

## Background

Globus pharyngeus is one of the most common diseases encountered in otolaryngology clinics. However, the treatment of globus pharyngeus still has some major problems with low cure rate and high recurrence. Similar patients may often be seen by gastroenterologists [1–3]. Manifestations of globus pharyngeus include, but are not limited to, laryngopharyngeal dryness, tightness, burning, obstruction, and foreign body sensation. However, these symptoms are not accompanied by sore throat or difficulty swallowing and/or breathing. The etiologies of globus pharyngeus are very complicated, including the diseases of the pharynx and its adjacent and/or distant organs, systemic disorders, mental factors and functional diseases. With the development of clinical technologies, a large number of studies have been performed [2, 3]. In these studies, researchers found a close relationship between globus pharyngeus and backflow of gastric contents into the throat. In 1987, Wiener et al. placed probes into the esophagus and on the top of the upper esophageal sphincter (UES) to detect pH over a course of 24 h using double-probe pH testing. They found that patients with globus pharyngeus had laryngopharyngeal acid reflux. In 1989, they once again used the similar methods to monitor pH over 24 h in 32 patients with globus pharyngeus. However, this time they reported that the symptoms and the acid reflux times of these patients were all different from gastroesophageal reflux disease (GERD), and esophagitis was hardly found. Since then, research on globus pharyngeus has been extensively performed [4]. Although the results were not completely consistent, laryngopharyngeal reflux (LPR) was officially adopted by the American Academy of Otolaryngology-Head and Neck Surgery in 2002 [5]. However, whether there was a close relationship between globus pharyngeus and laryngopharyngeal reflux and/or gastroesophageal reflux still left some doctors feeling confused. One study showed that abnormal laryngopharyngeal or esophageal reflux was not indicated by pH-impedance monitoring in some patients with suspected LPR refractory to proton pump inhibitors (PPIs) treatments. The results proved that LPR is unlikely in these patients [6]. It can be induced that globus pharyngeus patients with non-LPR (G-NR) also account for a certain proportion. At present, there is no report about the etiologies and influence factors of these patients. This study was performed to clarify the related factors of the symptoms of globus pharyngeus refractory to PPIs treatments and to learn more about G-NR so as to improve the curative effect.

## Methods

### Study subjects

This study recruited 42 “globus pharyngeus” patients (G group) and 38 non-globus pharyngeus patients (NG group) depending on whether they had abnormal laryngopharyngeal sensations. For “globus pharyngeus”, the Rome III definition was met except with the exception of that for reflux disease which was not objectively assessed. All cases were performed using High Resolution Manometry in Gastrointestinal Motility Suit, First Affiliated Hospital of Dalian Medical University from December 1, 2015 to May 1, 2016. The two groups were further divided into laryngopharyngeal non-reflux groups (G-NR group, NG-NR group) and laryngopharyngeal reflux groups (G-R group, NG-R group) according to the laryngopharyngeal Reflux Symptom Index (RSI) and the response to PPIs treatments [7]. This study was approved by the hospital ethics committee (No. LCKY2016–33), and all participants in the study signed the informed consent form.

### Inclusion and exclusion criteria

#### Inclusion and exclusion criteria of the G-NR group (*N* = 20)

##### Inclusion criteria

*a.* Conformed to Rome III criteria for globus [8]: 1*)* persistent or intermittent, non-painful sensation of a lump or foreign body in the throat; 2*)* occurrence of the sensation between meals; 3*)* absence of dysphagia or odynophagia; 4*)* absence of evidence that gastroesophageal reflux is the cause of the symptom (see b. d. e.); 5*)* absence of histopathology-based esophageal motility disorders. Criteria fulfilled for the last 3 months with symptom onset at least 6 months before diagnosis. *b*. no positive sign of inflammation, or only slight inflammation found through the electronic laryngoscopy and electronic gastroscopy; *c.* no history of drug use within the last 1 month; *d.* no response to double-dose of PPIs treatment for 8 ~ 12 weeks: no improvement in laryngopharyngeal symptoms; *e.* RSI scores < 13 [7].

##### Exclusion criteria

*a.* Accompanied by malignant tumor, immune system disease, metabolic disease, heart and lung diseases and severe local infection, etc.; *b.* unable to tolerate the esophageal pressure test performed by High Resolution Manometry.

#### Inclusion and exclusion criteria of the G-R group (*N* = 22)

##### Inclusion criteria

*a.* Abnormal feelings such as persistent or intermittent foreign body, formication sign, burning, tightening, muffled, narrow sense and sputum adhesion were exhibited in the oropharynx and suprasternal fossa, without the presence of dysphagia, sore throat or difficulty swallowing and/or breathing; *b.* no positive sign of inflammation, or only slight inflammation found through the electronic laryngoscopy and electronic gastroscopy; *c.* no history of drug use within the last 1 month. *d.* response to PPIs treatment. *e.* RSI scores ≥ 13 [7].

##### Exclusion criteria

Others are in agreement with those of the G-NR Group.

#### Inclusion and exclusion criteria of NG-NR group (*N* = 20)

##### Inclusion criteria

No symptoms of globus pharyngeus, such as laryngopharyngeal dryness, tightness, burning, obstruction or foreign body sensation; no sore throat, difficulty swallowing and/or breathing; but received the high resolution esophageal motility test for non-cardiac chest pain, i.e., pain or discomfort behind the sternum without any cardiopulmonary diseases. Other inclusion and exclusion criteria are in agreement with those of the G-NR Group.

#### Inclusion and exclusion criteria of NG-R group (*N* = 18)

##### Inclusion criteria

No symptoms of globus pharyngeus, but received esophageal pressure measurements because of the mild discomfort behind the sternum. Other inclusion and exclusion criteria are in agreement with those of the G-R Group.

### Questionnaires

The study participants completed the questionnaires under the guidance of a member of the study team who was present to ensure that the participants understood the forms clearly. However, the study member was prohibited from using any suggestive words regarding any of the questions.

#### General information

The general questionnaire included information about name, demographic data (age and gender), marriage, occupation, education, income, concomitant diseases, medications in use and family history.

#### RSI version

Patient information related to LPR symptoms was gathered through a questionnaire containing the validated version of RSI. The RSI score was applied as proposed by Belafsky et al., and LPR diagnosis was based on the RSI score ≥ 13 [7, 9]. A self-administered nine-item RSI was completed by the patients in less than 1 min. The score for each item ranged from 0 (none) to 5 (most severe) with a maximum total score of 45. The nine items included:1) hoarseness or a problem with your voice; 2) clearing your throat; 3) excessive throat mucus or postnasal drip; 4) difficulty swallowing food, liquids or pills; 5) coughing after eating or lying down; 6) breathing difficulties or choking episodes; 7) troublesome or annoying cough; 8) sensation of something sticking in your throat or a lump in your throat; 9) heartburn, chest pain, indigestion, or stomach acid reflux (Additional file 1).

#### Life exposure factors questionnaire [9–11]

Life exposure factors concerning behavioral characteristics probably related to globus pharyngeus were gathered through a questionnaire containing 17 items: smoking, alcohol, tea, coffee, chocolate, spicy food, greasy meal, fullness, high salt, night work, staying up late, fatigue, pressure, constipation, loneliness, anxiety and depression (Additional file 2).

### Esophageal motility measurement

High resolution manometry was performed using a 24-channel water-perfused catheter of 4.0 mm in diameter as used previous studies [12](Ningbo Maida Medical Device Inc., Ningbo, China). Side hole 1, which starts at the most distal point, was 5 cm from hole 2. Holes 2–7 were 1.0 cm from each other and holes 8–24 were 1.5 cm from each other. Once placed, it covered the entire length of the esophagus from the upper esophageal sphincter to the lower esophageal sphincter with an additional channel in the stomach. The data acquisition frequency was 20 Hz for each sensor. Computer analysis software (Video was recorded by MedSample360 at a rate of 20 frames/s and analyzed by MedView360) was used to assess various esophageal motility parameters.

The patients were asked to fast for 12 h for foods and 6 hours for water before the measurement. The high resolution esophageal motility test was performed in a supine position. Firstly, a 30-s period of basal recording was obtained after the appropriate placement of the catheter. Then, the patient was asked to swallow 5 ml of water and the swallow was repeated for a total of 10 times with a 30-s interval between consecutive swallows. Lastly, multiple rapid swallows (5 swallows of 2 ml of water with 2- to 3-s intervals) were performed. The resting pressure and residual pressure of the UES and low esophageal sphincter (LES), as well as the esophageal distal contractile integral (DCI), were recorded.

### Statistical analysis

All data were inputted into the SPSS 22.0 software package for processing. The *λ* [2] test was used for analysis of the correlation between the G group, the NG group and life exposure factors. The categorical data was shown using the percentage and analyzed by the *λ* [2] test. The measurement data are presented as mean ± standard deviation (x ± s). The Student’s *t* test was used for comparison between two groups. The significance level for all hypothesis testing (*p*-value) was 0.05.

## Results

### General data of patients

In the G group, the number of patients with non-laryngopharyngeal reflux symptoms (G-NR group) was 20/42, accounting for 48%; the number of patients with laryngopharyngeal reflux symptoms (G-R group) was 22/42, accounting for 52% (See Table 1). No statistical differences (*P* > 0.05) were noted in age among the 4 groups of patients (see Table 1).

**Table 1:** Patient information (gender and age)

	G-R Group	G-NR Group	NG-R Group	NG-NR Group
CASES	22	20	18	20
AGE (year)	49.85 ± 4.92	51.77 ± 5.09	52.41 ± 6.02	50.54 ± 4.21
SEX(male/female)	10/12	5/15	10/8	11/9

### Esophageal pressure results from HRM

#### Images of esophageal pressure measurements

**Fig. 1:**
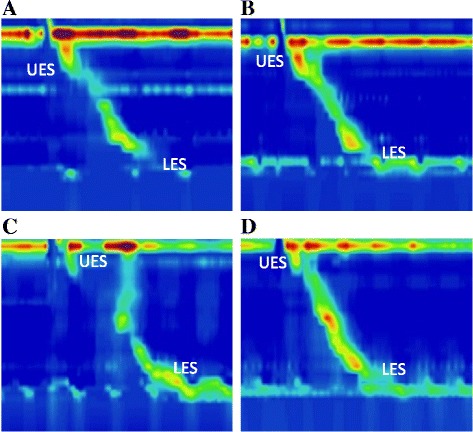
Typical high resolution esophageal manometric measurements in different groups of patients. **a**-**d** G-NR, G-R, NG-R and NG-NR

Typical images of esophageal motility measurements during a wet swallow in 4 different groups of patients are presented in Fig.  1. The top and bottom pressure bands reflect the UES and LES pressures before, during and after a wet swallow, respectively. The pressure tracings in between the UES and LES reflect esophageal body pressure changes during the swallow.

#### Analysis of UES

**Fig. 2:**
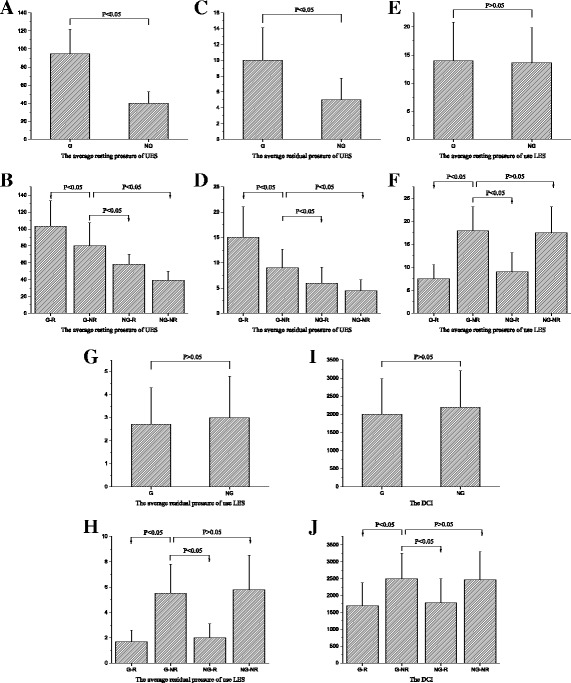
Esophageal manometric parameters in different groups of patients. **a** Average resting pressure of UES in patients with globus and patients without globus; **b** Average resting pressure of UES in 4 subgroups of patients; **c** Average residual pressure of UES in patients with globus and patients without globus; **d** Average residual pressure of UES in 4 subgroups of patients; **e** Average resting pressure of LES in patients with globus and patients without globus; **f** Average resting pressure of LES in 4 subgroups of patients; **g** Average residual pressure of LES in patients with globus and patients without globus; **h** Average residual pressure of LES in 4 subgroups of patients; **i** The DCI in patients with globus and patients without globus; **j** The DCI in 4 subgroups of patients

The average resting pressure of the UES in patients of the G group was higher than those in the NG group (*P* < 0.05, Fig. 2a). The resting UES pressure in the G-NR group was higher than that in the NG-R and NG-NR groups (*P* < 0.05), but lower than that in the G-R group (*P* < 0.05) (Fig. 2b). Similar differences were noted in the residual UES pressure among the groups: the average residual pressure of the UES in the G group was higher than that in the NG group (Fig. 2c); the average residual UES pressure in the G-NR group was higher than that in the NG-R and NG-NR groups (*P* < 0.05), but lower than that in the G-R group (*P* < 0.05) (Fig. 2d).

#### Analysis of LES

There was no statistical difference in the average resting pressure of the LES between the G group and the NG group (*P* > 0.05, Fig. 2e). The average resting pressure of the LES in the G-NR group was higher than that in the two G-R and NG-R reflux groups (*P* < 0.05) and showed no statistical difference with that in the NG-NR group (*P* > 0.05) (see Fig. 2f). The residual pressure of the LES exhibited similar results as the resting pressure of the LES among the groups (see Fig. 2g and 2h).

#### Analysis of DCI

There was no statistical difference in the DCI between the G group and the NG group (*P* > 0.05, Fig. 2i). The DCI in the G-NR group was higher than that in the G-R and NG-R groups (*P* < 0.05, Fig. 2j). No statistical difference was shown in the DCI between the G-NR group and the NG-NR group (*P* > 0.05, Fig. 2j).

### Comparison of age, sex and life exposures between groups

#### Age characteristics of G-NR patients

The average age of patients was 51 years in the G-NR group, 50 years in the G-R group, 52 years in the NG-R group, and 51 years in the NG-NR group (See Table 1). Both the median age and the mean age were within the middle range (45–60 years) described by the World Health Organization. The age difference was shown to be insignificant between the groups (*P* > 0.05). These results suggested that the middle age could be a risk factor for globus pharyngeus (including G-NR).

#### Analysis of sex and life exposure factors in G-NR patients

As shown in Tables 1 and 2, females accounted for the majority of patients in the G-NG group. So the gender female was taken as an investigative factor in the study. From the comparison of the results in G and NG of Table 2, we found that the incidences of smoking, alcohol consumption, high salt and anxiety were higher in the G group than in the NG group (*P* < 0.05). From the comparison of the results in G-NR and G-R of Table 2, we found that the incidences of smoking, alcohol consumption and anxiety were lower in the G-NR group than the G-R group (*P* < 0.05). From Table 1, we can see that the prevalence of globus pharyngeus was higher in female. However, from the comparison of the results in G-NR and NG-NR of Table 2, we found that the incidences of stress, smoking, alcohol consumption, consumption of high salt and anxiety were higher in the G-NR group compared with the NG-NR group (*P* < 0.05). Similarly, the prevalence of globus pharyngeus was higher in female than in male.

**Table 2:** Percentage of patients with different life exposure factors in each group

	G	NG	G-NR	G-R	NG-NR
Female*^#^^	64%	45%	75%	55%	45%
Depressed	8%	6%	5%	9%	5%
Anxiety*^#^^	27%	5%	15%	37%	5%
Loneliness	15%	11%	10%	18%	5%
Constipation	41%	36%	40%	41%	35%
Stress^^^	62%	56%	60%	64%	40%
Tiresome	27%	24%	25%	28%	30%
Stay up late	41%	40%	40%	41%	35%
Night shifts	10%	8%	5%	14%	10%
Spicy-food	36%	27%	35%	36%	40%
Fullness	12%	14%	15%	9%	20%
Oily-food	27%	24%	20%	28%	25%
Chocolate	22%	16%	20%	23%	25%
Coffee	41%	37%	45%	37%	50%
Strong tea	12%	11%	10%	14%	10%
Alcohol-drink*^^^	60%	35%	40%	64%	25%
High-salt*^#^^	60%	29%	60%	60%	35%
Smoking*^#^^	55%	19%	40%	69%	15%

Group G vs. Group NG: *P < 0.05; Group G-NR vs. Group G-R ^#^ *P* < 0.05; Group G-NR vs. Group NG-NR: ^^^ *P* < 0.05

## Discussion

In this study we have found that the average resting and residual pressures of the UES in the patients with globus pharyngeus without laryngopharyngeal reflux were higher than in the patients without globus pharyngeus. The patients with globus pharyngeus but no laryngopharyngeal reflux showed higher incidences of stress, smoking, alcohol-drinking, high-salt and anxiety than the patients without globus pharyngeus or laryngopharyngeal reflux.

The symptoms of globus pharyngeus are usually present with dry swallowing rather than swallowing food [13]. They may be intermittent or sustainable, often accompanied by other symptoms such as belching or chest tightness [14, 15]. The etiologies and mechanisms of globus pharyngeus have been unclear [16]. Some studies suggested that these symptoms might be associated with rhinitis, sinusitis, thyroid diseases, sore throat, GERD, gastroduodenal ulcer, iron deficiency anemia, psychological disorders, etc. [17, 18]. However, most researchers consider globus pharyngeus as a series of symptoms without organic diseases that are more like hysteria defined as a subjective feeling of throat discomfort.

Wada [19] reported that GERD could cause globus pharyngeus. LPR is believed to be attributed to abnormal motility of the UES and may often arise in daytime or in an upright position, especially during a physical exertion. If the PPI treatment is effective, the diagnosis of LPR can be considered. Otherwise further examinations are needed to determine the etiologies of globus pharyngeus [20–22].

The HRM system can provide clear, intuitive and accessible images and data of esophageal motility. However, there have been limited reports on the assessment of globus pharyngeus using the HRM techniques [23]. Our results showed that the resting and residual pressures of the UES in the globus pharyngeus group were higher than those of the non-globus pharyngeus group. These elevated UES pressure may be responsible to cause symptoms of globus via the vagal afferent pathway. The UES plays an important role in preventing reflux of esophageal contents through the throat and into the mouth. It was also found in this study that the resting and residual pressures of the UES in patients with globus and LPR were higher than those in patients with globus but no LPR; this elevation might be attributed to stimulation of the UES by the refluxed gastric acid [24].

The results of our study showed that patients with LPR with or without globus showed a reduced LES pressures in both resting and residual states, suggesting that symptoms of LPR might be caused by a low pressure of LES which is known to be a decisive factor in the occurrence of gastroesophageal reflux. Furthermore, the refluxed gastric acid could stimulate the ring pharyngeal muscle around the UES and result in spasms [24, 25]. The fact that patients with globus but no LPR exhibited normal LES pressure suggested that globus pharyngeus might not be associated with gastroesophageal reflux.

In this study, no significant difference was noted in DCI between the G-NR and NG-NR groups, indicating normal esophageal body motility in G-NR patients. However, the DCI in the G-R and NG-R groups was reduced in comparison with the NG-NR group, suggesting that abnormal esophageal body motility could occur in both reflux groups with and without globus pharyngeus. Most of previous studies were performed in the G-R group and yielded similar results [1, 26], whereas, little attention has been pain in the G-NR group. Weak distal esophageal smooth muscle contractions result in feeble peristalsis and decreases scavenging abilities of bolus and refluxed stomach contents. The residual food and refluxed acid may then stimulate the local esophagus, aggravating symptoms of laryngopharyngeal abnormal sensation [27, 28]. On contrary, the globus symptoms in the G-NR patients who showed normal DCI or peristalsis could not attributed to acid reflux or food/bolus retention. Taken together, the symptoms in the G-NR patients might be attributed mainly to the high pressure of the UES.

The life exposure factors play an important role in the occurrence and development and even treatment of diseases [11]. This study further explored relationships between age, sex, and life exposures in the G-NR. The mean age and median age of the patients in each group were all within the World Health Organization-specified middle-age range. Accordingly, the middle age could be regarded as a risk factor for globus pharyngeus, as in the cases of G-NR groups. Compared with the NG group, the G group had a higher incidence of smoking, alcohol consumption, eating high salt, and anxiety as well as a higher prevalence of globus pharyngeus in female. The incidence of globus pharyngeus was previously reported to be correlated with both anxiety and depression [29, 30]; however, the depression as a risk factor was not indicated in this study. Furthermore, we compared the G-NR group with the G-R group and found that the incidence of smoking, drinking and anxiety was lower in the G-NR patients. Combined with the above mentioned factors, the incidence of globus pharyngeus without reflux may be more likely to occur in middle-aged women, especially those with smoking habits, high rates of alcohol consumption and anxiety, but these habits have been shown to be more common in patients with reflux symptoms. Further, we compared the G-NR group with the NG-NR group and found a higher incidence of smoking, alcohol consumption, high salt consumption, stress and anxiety in the G-NR patients; in addition, the prevalence of globus pharyngeus was higher in female than male.

This study was limited in a number of issues: *a)* The number of patients recruited was limited, so a multi-center and large sample study could be performed. *b)* In our study, the NG-NR group acted as the control group only according to the RSI scores and responses to proton pump inhibitors (PPIs) treatments which may be used as objective parameters with low cost and high practicality [31, 32]. 24-h pH monitoring was not performed during the experiment to completely exclude the possibility of reflux [33].

## Conclusions

Globus pharyngeus without LPR may occur due to high UES pressure. Stress, smoking, alcoholic drinking, high salt consumption and anxiety may be its risk factors.

## Additional files

Additional file 1: The reflux symptom idex. (DOCX 68 kb)

Additional file 2: Life exposure factors questionnaire. (ZIP 45 kb)

## Abbreviations

DCI: Distal esophageal contraction integral
GERD: Gastroesophageal reflux disease
HRM: High Resolution Manometry
LES: Lower esophageal sphincter
LPR: Laryngopharyngeal reflux
LPRD: Laryngopharyngeal reflux disease
PPIs: Proton pump inhibitors
RSI: reflux symptom index
UES: Upper esophageal sphincter
WHO: the World Health Organization
