# Supplementary figures and images for: High-resolution manometry in patients with and without globus pharyngeus and/or symptoms of laryngopharyngeal reflux
Source: BMC Gastroenterol. 2017 Oct 23;17:109. doi: 10.1186/s12876-017-0666-x (PMC5654000; doi:10.1186/s12876-017-0666-x)

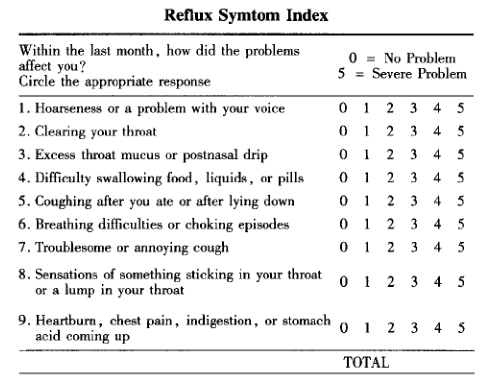

Supplement: Supplementary file 1 — The reflux symptom idex. (DOCX 68 kb) [file 12876_2017_666_MOESM1_ESM.docx]
