# Supplementary material for: High-resolution manometry in patients with and without globus pharyngeus and/or symptoms of laryngopharyngeal reflux
Source: BMC Gastroenterol. 2017 Oct 23;17:109. doi: 10.1186/s12876-017-0666-x (PMC5654000; doi:10.1186/s12876-017-0666-x)
Supplement: Supplementary file 2 — Life exposure factors questionnaire. (ZIP 45 kb) [file 12876_2017_666_MOESM2_ESM.zip › additional files-2-English.R3.doc]

Life exposure factors questionnaire

Note: in order to fully understand how about your health status and the life exposure factors in your daily life. Draw “√”before which you think much more appropriate. Thank you for your cooperation.

| Name |  | | Sex |  | Age |  | Degree of education | |  |
| --- | --- | --- | --- | --- | --- | --- | --- | --- | --- |
| Occupation |  | | Workplace |  | | | Telephone | |  |
| Height(cm) |  | | Weight(kg) |  | | | BMI(kg/m^2^) | |  |
| Marital Status | 口Unmarried 口Married 口Dissociation 口Widowed | | | | | | | | |
| Related factors | | Exposure frequency(nearly 6 months) | | | | | | Whether it will induce or aggravate heartburn and other discomfort | |
| depressed | | 口never 口occasionally 口often | | | | | | 口yes 口 no | |
| anxiety | | 口never 口occasionally 口often | | | | | | 口yes 口 no | |
| loneliness | | 口never 口occasionally 口often | | | | | | 口yes 口 no | |
| constipation | | 口never 口occasionally 口often | | | | | | 口yes 口 no | |
| stress | | 口never 口occasionally 口often | | | | | | 口yes 口 no | |
| tiresome | | 口never 口occasionally 口often | | | | | | 口yes 口 no | |
| stay up late | | 口never 口occasionally 口often | | | | | | 口yes 口 no | |
| night shifts | | 口never 口occasionally 口often | | | | | | 口yes 口 no | |
| spicy-food | | 口never 口occasionally 口often | | | | | | 口yes 口 no | |
| fullness | | 口never 口occasionally 口often | | | | | | 口yes 口 no | |
| oily-food | | 口never 口occasionally 口often | | | | | | 口yes 口 no | |
| chocolate | | 口never 口occasionally 口often | | | | | | 口yes 口 no | |
| coffee | | 口never 口occasionally 口often | | | | | | 口yes 口 no | |
| strong tea | | 口never 口occasionally 口often | | | | | | 口yes 口 no | |
| alcohol-drink | | 口never 口occasionally 口often（≥80g/day) | | | | | | 口yes 口 no | |
| high-salt | | 口never 口occasionally 口often | | | | | | 口yes 口 no | |
| smoking | | 口never 口occasionally 口often（≥2/day) | | | | | | 口yes 口 no | |
| Remarks occasionally：less than once a week  often：almost every day or more than once a week | | | | | | | | | |
